# Ninety-day repeated dose toxicity of Ashwagandha (Withania somnifera) root extract in Wistar rats

**DOI:** 10.1016/j.toxrep.2023.09.004

**Published:** 2023-09-09

**Authors:** P. Kalaivani, R. Siva, V. Gayathri, Deepak Langade

**Affiliations:** a Centre For Toxicology and Developmental Research (CEFTE), Sri Ramachandra Institute of Higher Education and Research, Chennai, Tamil Nadu, India; b Dr. D. Y. Patil University School of Medicine, Navi Mumbai, Maharashtra, India

**Keywords:** Ashwagandha root extract, Toxicity, Repeat dose

## Abstract

Many pharmacological studies have been carried out to describe multiple biological properties of Ashwagandha (Withania somnifera) and the additional safety information on repeated dose toxicity is limited. Therefore, the aim of this study was to obtain safety data for KSM-66 Ashwagandha Root Extract (ARE) through repeated-dose toxicity in Wistar rats according to the Organisation for Economic Co-operation and Development (OECD) test guideline (TG 408). ARE was orally administered to rats at doses of 0, 500, 1000, and 2000 mg/kg body weight/day for 90-day and reversibility of effects of 0 and 2000 mg/kg body weight/day was assessed for 14 days. All the animals from treated, control, recovery control and recovery groups were observed for clinical signs of toxicity once daily, detailed clinical examination every week after dosing and before necropsy day. Mortality/Morbidity was observed twice daily. In addition, observations were noted in the detailed sensory reactivity, functional assessments, body weight, food consumption, ophthalmological examination, hematological parameters, biochemical parameters, organ weights, histopathological findings. The present results show that the no observed adverse effect level (NOAEL) of KSM-66 Ashwagandha Root Extract was considered to be 2000 mg/kg body weight/day in rats after repeated oral administration for 90-day under the present study conditions.

## Highlights

• Studies on Ashwagandha’s safety and repeated dose toxicity data are limited.
• Repeated-dose toxicity study conducted on Ashwagandha Root Extract (ARE) in rats.
• ARE test doses were 0, 500, 1000, and 2000 mg/kg body weight/day for 90 days.
• No observed adverse effect level determined as 2000 mg/kg body weight/day in rats.

## Graphical Abstract

**Figure:**
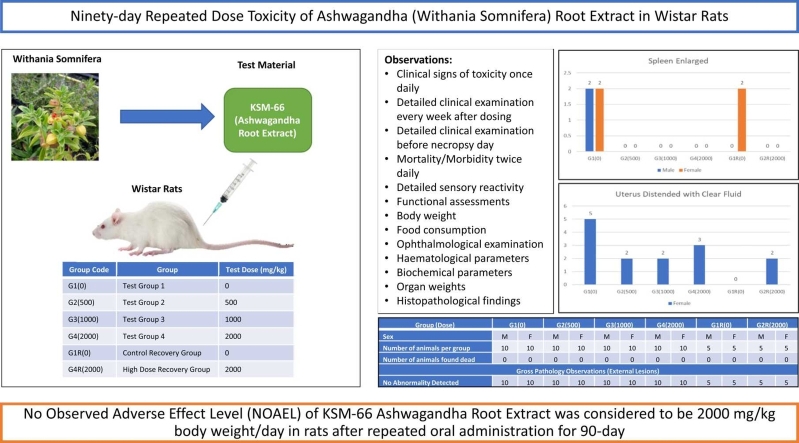

## 1 Introduction

For over four millennia, plants have been utilized as medicinal remedies [1]. More than 80,000 of the 250,000 species of flowering plants have been reported to be used medicinally by human civilizations. This number may be larger because knowledge of native applications for plants as remedies has largely been passed down verbally from one generation to the next and has remained largely unreported. Due to the secrecy and reluctance of some practitioners to divulge enough information, part of the traditional wisdom may have been lost. History books, archives, old papers, archeological discoveries, and pharmacopeias contains lot of information on the history of using plant ingredients as medicines. Humans have used plants as remedies at least since the Middle Paleolithic, or around 60,000 years ago, according to fossil records [2].

Plants have always been a rich source of natural products and historically provided many major new drugs [2]. Through the presence of biologically active phytochemicals, they have provided treatment for a vast range of illnesses and continue to be highly sought after by the pharmaceutical industry [3]. Their therapeutic properties have been well-documented in treating both communicable (viral and bacterial) and non-communicable ailments, such as cancer, diabetes, autoimmune disorders, and more [4], [5], [6]. The scientific study of plant-based medicine commenced in a systematic manner during the 1950 s [2]. According to estimates by the World Health Organization (WHO), around 80% of the global population relies on herbal drugs for health benefits [7].

The greatest repository of medicinally valuable plants among all ancient cultures is found in India. Withania somnifera (Ashwagandha), also referred to as "Indian Ginseng" or "Indian Winter cherry," is one such essential medicinal plant and has been used as an Ayurvedic rasayana since ancient culture [8]. In biological systems, it shows pleotropic effects that include, among others, neuroprotective, anti-inflammatory, immune-modulatory, and antitumor activity [9]. The root of Ashwagandha, which includes numerous biologically active ingredients like alkaloids (isopelletierine, anahygrine, etc.), saponins, and steroidal lactones, is the most widely used part of this plant in Ayurvedic formulations (withanolides and withaferins) [10], [11].

Many pharmacological studies have been carried out to describe multiple biological properties of W. somnifera. However, there is little information on repeated dose toxicity. Therefore, we investigated the potential repeated dose toxicity, providing useful information for assessing the toxicological relevance of W. somnifera. This study was conducted according to Organisation for Economic Co-operation and Development (OECD) test guideline 408 repeated dose 90-day oral toxicity study in rodents (adopted: 25th June 2018) to determine the possible health hazards likely to arise from repeated oral administration of the test item “KSM-66 Ashwagandha Root Extract” for a period of 90 days in Wistar rats. The study also focused on determining No Observed Adverse Effect Level (NOAEL) and to assess the reversibility, persistence and delayed toxic effects of “KSM-66 Ashwagandha Root Extract”.

## 2 Materials and methods

### 2.1 Test material and preparation

KSM-66 Ashwagandha is a root extract of ashwagandha manufactured using an aqueous-based extraction process. It is slightly hygroscopic and yellowish-brown in color. It is standardized to > 5% of total withanolide content, consisting mainly of Withastramonolide A, Withanoside IV, Withanolide A, and Withanone. It also consists of < 0.1% of withaferin A. KSM-66 Ashwagandha Root Extract (ARE) was provided by Shri Kartikeya Pharma (Telangana, India). The test item was suspendable in 0.1% Carboxy Methyl Cellulose (CMC) as per the datasheet hence same was used as vehicle. Suspendability of the test item in vehicle was ensured a day prior to dosing. Homogeneity was maintained by constantly stirring with a glass rod during test item administration.

The dose formulation was prepared freshly in 0.1% CMC (vehicle) prior to administration. Required quantity of test item was weighed and grounded using mortar and pestle. The required volume of vehicle was added in the mortar and stirred well using a pestle to make a fine pasty liquid and then it was transferred to a measuring cylinder. The required volume of the vehicle was further added to the measuring cylinder to make up the final volume of formulation and transferred to a labeled beaker for dosing. The prepared formulation was mixed or stirred using a glass rod constantly to maintain uniformity and homogeneity of the test item after preparation until dosing.

### 2.2 Animal husbandry and maintenance

Fifty Wistar Rats of each sex were obtained from In-vivo Biosciences (Bengaluru, India) and used following acclimatization period. The median age of these rats was 9 weeks (8–10 weeks) at the start of dosing. The animals were housed in a room maintained at a temperature of 19.11–22.18 C and a relative humidity of 52.7–68.6%, with artificial lighting 12 h and 10–15 air changes per hr. Only healthy animals were included in the study. Animals were housed in groups in polypropylene cages covered with stainless steel grid top. Autoclaved paddy husk was used as bedding material. Cages were changed on alternative days and cage grills were changed once a week. Animals were fed with laboratory rodent pelleted feed procured from approved vendor, ad libitum except on the day of fasting. Potable UV irradiated purified water was provided ad libitum in autoclaved bottles. Water bottles were changed daily.

### 2.3 Selection of doses and treatment

The doses were selected as Low dose (500 mg/kg), Mid dose (1000 mg/kg) and High dose (2000 mg/kg) respectively based on the two weeks dose range finding study of KSM-66 Ashwagandha Root Extract. The route of administration was proposed as oral based on regulatory guideline. Dose formulation was prepared freshly in 0.1% CMC (vehicle) prior to administration. The study employed three graduated doses of test item (low, mid, and high dose), one recovery high dose, and two control groups (one control and one control recovery). Animals (10 animals/sex/group) and (5 animals/sex/group) were dose as mentioned below (Table 1).

**Table 1:** Groups for 90-day repeated oral toxicity study.

Group No.	Treatment	Dose (mg/kg)	Male		Female	
			No. of Animals	Animal No.	No. of Animals	Animal No.
G1	Control (Vehicle)	0	10	1–10	10	51–60
G2	Low dose	500	10	11–20	10	61–70
G3	Mid dose	1000	10	21–30	10	71–80
G4	High dose	2000	10	31–40	10	81–90
G1R	Control recovery	0	5	41–45	5	91–95
G4R	High dose recovery	2000	5	46–50	5	96–100

### 2.4 Mortality, morbidity and clinical signs of toxicity

Animals from all groups were observed for mortality and morbidity twice daily from acclimatization, till the day of necropsy. General clinical examinations of animals from all groups were carried out once daily (after the day's dosing) until the day of necropsy. A detailed clinical examination was performed prior to the first dosing and then once a week until the necropsy.

### 2.5 Body weight

Individual body weight of the animals was recorded on the first day and every week of treatment and at the day of necropsy. The body weight changes for all the animals were calculated.

### 2.6 Feed consumption

Cage wise feed consumption of animals was recorded daily from the day first of dosing (day 1), till the day of necropsy (day 90 and day 104), except on the day of overnight fasting. The measurement of average feed consumption per animal was calculated per week.

### 2.7 Ophthalmological examination

Ophthalmological examination was performed in animals using ophthalmoscope once before test item exposure (day 0) and day before necropsy (day 90 Phase 1 & 2 and day 104 Phase 3).

### 2.8 Functional assessment

The functional observation parameters such as sensory reactivity, grip strength and motor activity were determined on 12th week for main group (Phase-1 & 2) and 14th week (Phase-3) for recovery group of the experimental period for all animals.

### 2.9 Hematology

Whole blood was collected through retro orbital puncture from all the groups using isoflurane anesthesia at the end of treatment period (day 91 and 105). Prior to blood collection, the animals were fasted overnight with free access to water. Whole blood was used for the analysis of hematology parameters such as Total Leucocytes Count, Differential Count, Leucocyte - Neutrophil, Lymphocytes, Monocytes, Eosinophil and Basophil, Total Erythrocyte Count, Haemoglobin, Haematocrit, and Platelet Count, Mean Corpuscular Volume, Mean Corpuscular Haemoglobin, Mean Corpuscular Concentration. Additionally, the blood clotting time and total reticulocyte count were examined manually.

### 2.10 Biochemistry

Serum sample was collected and analyzed for Alanine aminotransferase, Aspartate aminotransferase, Alkaline Phosphatase, Total Cholesterol, HDL Cholesterol, LDL Cholesterol, Creatinine, Urea, Blood Urea Nitrogen (Calculated from Urea), Glucose, Phosphorus, Total Bilirubin, Total Protein, Albumin and Triglycerides. In addition, thyroid hormones (T3, T4) and thyroid stimulating hormone (TSH) were measured.

### 2.11 Pathology, organ weight and histopathology

All animals were fasted overnight on day 90 and 104; necropsied on day 91 and 105 respectively, using carbon dioxide in the euthanasia chamber and were subjected to detailed gross necropsy. Gross post-mortem examinations of the external orifices and the organs in the cranial, thoracic, and abdominal cavities of the sacrificed rats were performed. Absolute and relative weights (organ to body weight ratio) of organs were measured; these included the brain, pituitary gland, thyroid with parathyroids, thymus, spleen, liver, adrenal glands, kidneys, epididymides, prostate, testes, ovaries and uterus with cervix. Vaginal cytology was observed in female animals of all the groups on day 91 and 105. Histopathology was carried out on the preserved organs and tissues of all animals in the control and high dose groups.

### 2.12 Statistical analysis

Statistical analysis was performed to analyze the mean differences between test item and control. The statistical analysis was performed using one-way analysis of variance (ANOVA) followed by Dunnett’s post hoc using Sigma Plot 12.3 software. Each group mean was presented along with Standard Deviation and the number of animals/observations (N). The “p” value ≤ 0.05 was fixed as significance criterion. Data, including body weight along with body weight changes, feed consumption, Hematology, Biochemistry and organ weight (absolute and relative) were subjected to statistical analysis.

## 3 Results and discussion

### 3.1 Mortality, morbidity and clinical signs of toxicity

No overt signs of toxicity were observed in any animals treated with the test substance. No mortality was observed during the treatment or recovery period. Vaginal cytology was observed in female animals of all the groups without any test item related effects observed on day 91 and 105. Our repeated dose toxicity findings were similar to an acute and sub-acute oral toxicity study with hydroalcoholic extract of Withania somnifera roots which showed no evidence of toxic effect or mortality in Wistar rats. Similarly, hydroalcoholic extract of Withania somnifera was found to be safe in sub-acute toxicity study involving rats and mice. However, the content of Withaferin A in these hydroalcoholic extracts were not reported [12], [13].

### 3.2 Body weight

In females, statistically significant changes were observed from day 1 and 8 (G4R) when compared with control group (G1R) during the experimental period (p ≤ 0.05) (Table 2).

**Table 2:** Summary of body weight in female rats.

Days	1	8	15	22	29	36	43	50	57	64	71	78	85	92	99	105
Groups	Mean (SD)	Mean (SD)	Mean (SD)	Mean (SD)	Mean (SD)	Mean (SD)	Mean (SD)	Mean (SD)	Mean (SD)	Mean (SD)	Mean (SD)	Mean (SD)	Mean (SD)	Mean (SD)	Mean (SD)	Mean (SD)
G1(0)	164.47 (16.98)	173.3 (20.35)	187.29 (20.83)	199.17 (17.01)	208.75 (13.19)	215.56 (16.55)	219.02 (13.89)	222.8 (12.32)	232.2 (16.04)	236.02 (17.98)	241.78 (11.49)	241.3 (11.42)	243.32 (17.21)	228.16 (12.88)	NA	NA
G2(500)	167.01 (12.05)	179.34 (15.83)	193.92 (16.19)	209.81 (15.94)	220.08 (17.81)	227.74 (18.35)	231.79 (19.25)	234.34 (20)	240.74 (20.86)	244.91 (22.46)	248.09 (21.75)	253.61 (23.5)	246.26 (30.24)	225.5 (23.84)	NA	NA
G3(1000)	161.49 (9.37)	168.39 (13.44)	189.11 (16.8)	201.32 (14.62)	210.3 (14.1)	219.53 (17.75)	223.16 (16.32)	225.89 (16.24)	233.21 (20.19)	236.61 (21.27)	241.96 (16.94)	246.51 (21.16)	251.83 (24.28)	232.84 (20.9)	NA	NA
G4(2000)	159.99 (11.52)	171.36 (14.18)	189.99 (9.72)	206.08 (14.56)	216.74 (17.63)	226.36 (18.2)	231.93 (20.28)	235.17 (20.1)	242.83 (20)	248.84 (20.88)	251.19 (22.21)	249.08 (33.56)	254.49 (35.26)	230.83 (27.78)	NA	NA
G1R(0)	165.4 (7.44)	183.31 (7.3)	200.03 (6.72)	212.44 (12.77)	215.16 (12.58)	227.14 (13.92)	233.51 (15.97)	237.8 (18.79)	248.41 (22.64)	253.06 (25.53)	253.9 (24.93)	266.99 (23.1)	272.51 (25.76)	261.6 (22.18)	256.5 (22.09)	246.68 (20.66)
G4R(2000)	179.81 (6.73)	196.74 (4.36)	206.89 (6.61)	219.38 (8.69)	222.94 (6.81)	237.96 (5.9)	239.63 (6.4)	240.27 (6.85)	255 (7.74)	252.93 (12.51)	253.56 (12.53)	268.83 (18.26)	280.36 (14.51)	266.44 (13.07)	268.44 (11.04)	257.61 (7.39)

G1(0): Group 1 (Test Dose: 0 mg/kg), G2(500): Group 2 (Test Dose: 500 mg/kg), G3(1000): Group 3 (Test Dose: 1000 mg/kg), G4(2000): Group 4 (Test Dose: 2000 mg/kg), G1R(0): Control Recovery Group (Test Dose: 0 mg/kg), G4R(2000): High Dose Recovery Group (Test Dose: 2000 mg/kg). NA: Not Applicable. SD: Standard Deviation.

### 3.3 Body weight changes (Recovery group)

**Fig. 1:**
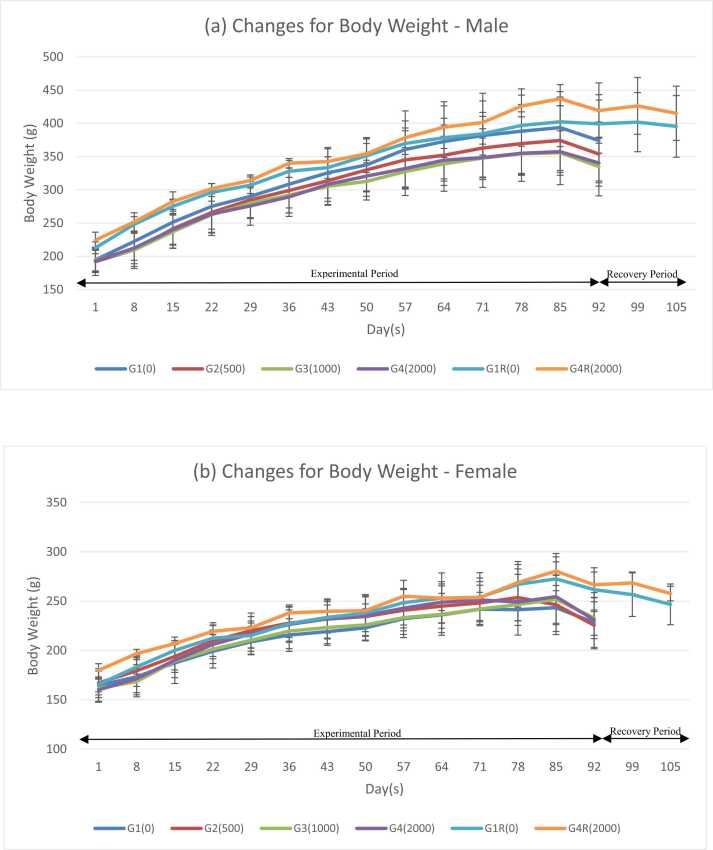
Changes for body weight: (a) Body weight (male); (b) Body weight (female).

**Fig. 2:**
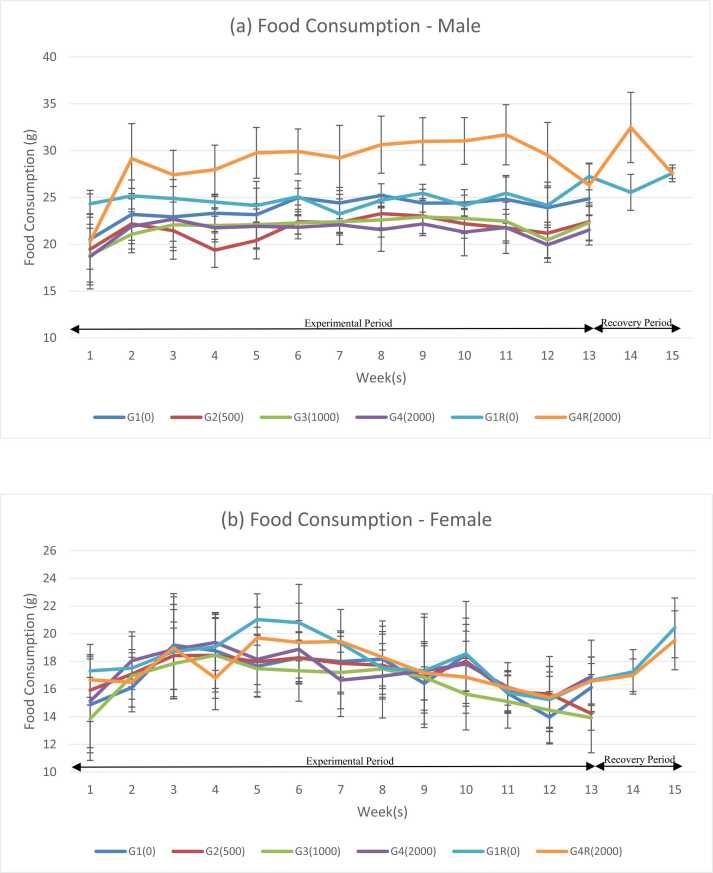
Changes for food consumptions: (a) Food consumptions (male); (b) food consumptions (female).

In males, statistically significant changes were observed in from day 78 and 85 (G4R) when compared with G1R group throughout the experimental period (p ≤ 0.05). While there was statistically significant decrease and, increase in female G4R group from day 8 to day 15, and 29–36 (p < 0.05) when compared with the control groups in females. These statistically significant changes could not be attributed to the test item related effect as were observed in both sexes but were not consistent and also observed in other group of animals during the observation period. These findings were similar to acute and sub-acute oral toxicity studies of the hydroalcoholic extract of Withania somnifera roots in Wistar rats [12]. Fig. 1, Fig. 2.

### 3.4 Feed Consumption

Weekly feed consumption of all treatment groups G2, G3 and G4 were comparable with control group G1 throughout the experimental period.

#### 3.4.1 Main group

In males, statistically significant changes were observed from 4th week to 13th week except for 9th week in G2 group; from 6th week to 13th week, except for 10th week G3 group, from 6th week to 13th week in G4 group when compared with control group (G1) throughout the experimental period (p ≤ 0.05). In females, statistically significant changes were observed in 10th week for G3 group and 2nd week for G4 group when compared with the control group (G1) throughout the experimental period and the computed (p ≤ 0.05).

#### 3.4.2 Recovery group

In males, statistically significant changes were observed in 1st week to 2nd week, 4th week to 12th week and 14th week whereas it was not observed in 3rd week and 13th week when compared with the control group (G1R) during the experimental period (p ≤ 0.05).

In recovery group female, statistically significant changes were observed in 4th week and 6th week G4R when compared with the control group (G1R) throughout the experimental period (p ≤ 0.05).

This statistically significant changes observed in feed consumption were minimal, transient and considered to be inconsistent among the treatment groups. Hence this could not be attributed to the test item related effects.

### 3.5 Ophthalmological examination

Both the eyes of all animals in the main and recovery group appeared normal before the start of experiment till necropsy. Thus, no test item related changes were observed in ophthalmoscope examination.

### 3.6 Sensory reactivity and motor activity

No test item related changes in sensory reactivity and motor activity were observed in control and treated group of animals except female recovery group. In female recovery group, statistically significant changes were noted in G4R of grip strength and motor activity when compared with G1R.

### 3.7 Hematology and biochemistry

An acute and sub-acute administration of standardized Withania somnifera extract investigated the potential adverse effects in rats and observed increase in hemoglobin and white blood cell counts and decrease in reticulocyte count and prothrombin time [14]. However, there were no test item-related changes observed in hematology or biochemical parameters in any of the treated groups when compared with control group in our study (Table 3, Table 4).

**Table 3:** Hematological parameters.

Parameters	Group (Dose)	G1 (0)	G2 (500)	G3 (1000)	G4 (2000)	G1R (0)	G4R (2000)
	Sex/Day	Day 91	Day 91	Day 91	Day 91	Day 105	Day 105
		*Mean (SD)*	*Mean (SD)*	*Mean (SD)*	*Mean (SD)*	*Mean (SD)*	*Mean (SD)*
Total Leucocyte Count (10^3 cells/μL)	Male	13.69 (2.55)	14.21 (2.27)	13.78 (2.56)	13.11 (2.29)	12.91 (3.13)	14.59 (2.20)
	Female	10.98 (2.99)	11.40 (2.82)	11.03 (3.07)	11.96 (3.13)	9.22 (3.46)	10.35 (2.84)
Neutrophils (%)	Male	16.43 (2.08)	18.87 (6.67)	18.03 (4.67)	21.72 (6.93)	19.98 (2.28)	17.48 (3.77)
	Female	23.25 (6.60)	18.97 (3.34)	21.05 (6.58)	25.13 (7.48)	23.56 (11.04)	18.18 (5.71)
Lymphocytes (%)	Male	73.96 (3.39)	72.68 (9.17)	73.20 (5.85)	69.15 (9.03)	69.86 (3.43)	70.48 (6.19)
	Female	66.66 (8.52)	74.61 (4.28)	70.03 (8.45)	66.11 (9.61)	67.32 (11.45)	72.42 (7.07)
Monocytes (%)	Male	6.98 (2.35)	6.12 (2.41)	6.66 (1.71)	6.78 (2.42)	7.04 (1.73)	8.96 (2.05)
	Female	6.58 (1.76)	6.22 (1.71)	5.98 (1.64)	6.25 (2.62)	6.66 (2.01)	6.90 (1.56)
Eosinophils (%)	Male	1.76 (0.54)	1.39 (0.62)	1.21 (0.43)	1.55 (0.58)	2.18 (0.42)	1.98 (0.72)
	Female	2.42 (1.83)	1.49 (0.49)	1.76 (1.93)	1.56 (0.60)	1.36 (0.61)	1.18 (0.18)
Basophils (%)	Male	0.87 (0.13)	0.94 (0.50)	0.90 (0.19)	0.80 (0.27)	0.94 (0.36)	0.76 (0.30)
	Female	1.09 (0.48)	0.71 (0.36)	1.18 (0.47)	0.95 (0.43)	1.1 (0.70)	1.32 (0.52)
Total erythrocyte count (10^6 cells/μL)	Male	8.73 (0.86)	8.12 (1.30)	8.08 (0.51)	8.40 (0.97)	8.96 (1.05)	9.00 (0.67)
	Female	7.64 (1.31)	7.38 (1.15)	7.86 (0.90)	7.66 (0.80)	7.44 (1.18)	7.44 (1.03)
Hemoglobin concentration (g/dL)	Male	15.03 (1.51)	14.62 (1.82)	14.61 (0.92)	14.90 (1.34)	15.86 (1.05)	16.00 (0.60)
	Female	14.55 (1.82)	13.91 (1.74)	14.25 (1.45)	14.48 (1.19)	14.26 (1.86)	14.5 (1.30)
Hematocrit (%)	Male	45.61 (4.78)	43.12 (5.25)	42.61 (2.67)	44.16 (4.78)	46.08 (4.56)	45.78 (1.96)
	Female	43.91 (4.49)	41.21 (5.78)	42.28 (4.90)	43.06 (4.16)	41.96 (4.81)	41.58 (4.52)
Platelet (10^3 cells/μL)	Male	982.60 (137.90)	958.70 (162.24)	944.80 (80.56)	1025.70 (111.42)	991.40 (200.58)	1063.20 (160.96)
	Female	862.40 (244.66)	976.30 (199.83)	1090.10 (158.91)	1034.50 (184.38)	826.60 (197.40)	926.80 (164.58)
Mean corpuscular volume (fL)	Male	52.23 (1.7)	53.54 (3.69)	52.73 (1.05)	52.72 (2.59)	51.50 (1.78)	50.94 (1.78)
	Female	58.39 (6.81)	56.01 (3.00)	53.81 (1.88)	56.25 (1.60)	56.74 (4.10)	56.20 (3.87)
Mean corpuscular hemoglobin (pg)	Male	17.21 (0.5)	18.13 (1.20)	17.34 (0.83)	17.81 (0.76)	17.80 (1.04)	17.80 (0.71)
	Female	19.23 (1.45)	18.94 (1.23)	18.18 (0.68)	18.95 (0.58)	19.26 (0.77)	19.58 (1.01)
Mean corpuscular hemoglobin concentration (g/dL)	Male	33.64 (1.37)	33.60 (0.47)	34.27 (0.97)	33.74 (0.71)	34.52 (1.46)	34.92 (0.23)
	Female	33.12 (1.82)	33.83 (1.00)	33.79 (0.66)	33.69 (0.66)	34.02 (1.72)	34.92 (1.73)
Clotting Time (sec)	Male	104.70 (11.36)	97.40 (9.34)	94.50 (12.99)	93.60 (13.38)	94.40 (3.51)	95.60 (2.3)
	Female	94.20 (8.97)	95.50 (13.34)	100.10 (9.64)	103.8 (12.46)	98.80 (2.95)	102.40 (2.97)
Reticulocyte (%)	Male	1.90 (0.74)	1.90 (0.74)	2.10 (0.88)	2.10 (0.74)	2.00 (0.71)	2.00 (0.71)
	Female	2.10 (0.74)	2.10 (0.74)	1.90 (0.88)	2.10 (0.88)	2.00 (0.71)	2.00 (0.71)

G1(0): Group 1 (Test Dose: 0 mg/kg), G2(500): Group 2 (Test Dose: 500 mg/kg), G3(1000): Group 3 (Test Dose: 1000 mg/kg), G4(2000): Group 4 (Test Dose: 2000 mg/kg), G1R(0): Control Recovery Group (Test Dose: 0 mg/kg), G4R(2000): High Dose Recovery Group (Test Dose: 2000 mg/kg). SD: Standard Deviation.

**Table 4:** Biochemistry parameters.

Parameters	Group (Dose)	G1 (0)	G2 (500)	G3 (1000)	G4 (2000)	G1R (0)	G4R (2000)
	Sex/Day	Day 91	Day 91	Day 91	Day 91	Day 105	Day 105
		*Mean (SD)*	*Mean (SD)*	*Mean (SD)*	*Mean (SD)*	*Mean (SD)*	*Mean (SD)*
Glucose (mg/dL)	Male	77.70 (11.58)	77.50 (9.70)	77.60 (14.62)	77.30 (17.28)	75.20 (10.64)	77.00 (6.96)
	Female	61.00 (5.83)	60.80 (6.14)	61.00 (9.79)	63.00 (9.82)	76.20 (12.79)	73.00 (9.51)
Total cholesterol (mg/dL)	Male	49.80 (7.89)	55.90 (12.39)	54.10 (7.61)	50.80 (12.05)	42.00 (5.66)	42.00 (8.31)
	Female	52.00 (7.92)	60.60 (12.46)	54.90 (10.35)	60.50 (14.28)	58.00 (12.59)	72.20 (8.47)
Triglycerides (mg/dL)	Male	94.60 (12.21)	94.70 (9.86)	94.50 (15.08)	96.30 (10.09)	80.80 (12.93)	81.20 (13.27)
	Female	92.30 (13.15)	97.10 (7.03)	100.60 (9.80)	94.50 (9.98)	79.00 (7.48)	91.60 (19.55)
Low-density lipoprotein (mg/dL)	Male	13.38 (1.69)	11.95 (1.51)	12.82 (1.55)	13.21 (1.60)	16.16 (7.94)	14.60 (4.38)
	Female	12.88 (1.55)	12.81 (1.61)	13.26 (1.69)	12.47 (1.52)	13.26 (6.84)	11.80 (2.53)
High-density lipoprotein (mg/dL)	Male	23.18 (2.09)	23.64 (2.34)	23.14 (2.03)	23.09 (2.23)	32.60 (4.75)	34.50 (3.72)
	Female	22.76 (2.49)	23.37 (2.20)	23.28 (1.70)	24.49 (2.49)	48.76 (7.76)	44.96 (4.35)
Total bilirubin (mg/dL)	Male	0.30 (0.12)	0.30 (0.11)	0.30 (0.12)	0.30 (0.11)	0.32 (0.13)	0.30 (0.07)
	Female	0.42 (0.12)	0.36 (0.20)	0.35 (0.20)	0.33 (0.13)	0.44 (0.17)	0.48 (0.13)
Phosphorus (mg/dL)	Male	8.45 (0.90)	8.14 (1.25)	8.09 (1.46)	7.80 (1.37)	8.45 (0.96)	7.62 (0.82)
	Female	6.38 (4.48)	6.58 (4.34)	6.68 (4.61)	6.78 (4.30)	7.11 (0.84)	9.68 (3.50)
Urea (mg/dL)	Male	43.10 (7.08)	44.70 (4.90)	44.00 (3.94)	44.30 (5.72)	28.40 (1.95)	29.20 (3.56)
	Female	48.40 (7.81)	49.70 (7.62)	47.10 (6.37)	46.10 (6.89)	33.60 (6.02)	33.60 (3.58)
Blood urea nitrogen (mg/dL)	Male	20.14 (3.31)	20.89 (2.29)	20.56 (1.84)	20.70 (2.67)	13.27 (0.91)	13.64 (1.67)
	Female	22.62 (3.65)	23.22 (3.56)	22.01 (2.98)	21.54 (3.22)	15.70 (2.82)	15.70 (1.67)
Creatinine (mg/dL)	Male	0.54 (0.06)	0.56 (0.10)	0.55 (0.07)	0.57 (0.07)	0.51 (0.05)	0.50 (0.05)
	Female	0.63 (0.11)	0.64 (0.12)	0.62 (0.12)	0.56 (0.06)	0.54 (0.10)	0.55 (0.03)
Total protein (g/dL)	Male	7.45 (0.74)	7.47 (0.49)	7.43 (0.34)	7.45 (0.42)	7.41 (0.23)	7.26 (0.22)
	Female	8.05 (0.30)	7.90 (0.61)	7.80 (0.49)	7.94 (0.37)	8.08 (0.21)	7.92 (0.33)
Albumin (g/dL)	Male	3.30 (0.29)	3.39 (0.20)	3.49 (0.14)	3.41 (0.22)	3.36 (0.28)	3.16 (0.13)
	Female	3.61 (0.38)	3.63 (0.24)	3.66 (0.42)	3.67 (0.31)	3.48 (0.20)	3.35 (0.21)
Alanine aminotransferase (U/L)	Male	71.30 (13.57)	75.40 (15.78)	74.30 (13.77)	82.40 (11.02)	52.20 (8.58)	49.20 (3.83)
	Female	74.50 (13.06)	80.90 (26.47)	71.00 (24.11)	64.00 (18.68)	55.40 (7.89)	52.80 (6.18)
Aspartate aminotransferase (U/L)	Male	202.70 (42.91)	203.70 (37.40)	204.00 (27.72)	205.80 (41.65)	140.40 (14.31)	126.60 (17.92)
	Female	213.70 (33.45)	181.10 (63.75)	182.40 (60.76)	170.20 (54.92)	120.80 (12.91)	126.20 (14.41)
Alkaline phosphatase (U/L)	Male	129.60 (43.26)	142.50 (36.59)	146.40 (29.57)	124.40 (65.78)	122.60 (37.26)	128.00 (24.08)
	Female	97.40 (23.16)	105.20 (39.30)	95.40 (21.27)	119.30 (28.43)	69.40 (13.45)	63.80 (18.62)
Sodium (mmol/L)	Male	141.00 (0.82)	140.60 (1.43)	140.90 (1.10)	140.30 (1.34)	139.60 (1.52)	140.40 (0.55)
	Female	141.40 (0.52)	141.70 (0.48)	141.30 (0.48)	141.10 (0.88)	141.00 (0.00)	140.60 (0.89)
Potassium (mmol/L)	Male	4.87 (0.28)	4.92 (0.24)	4.89 (0.24)	4.91 (0.22)	4.74 (0.09)	4.64 (0.05)
	Female	3.88 (0.51)	3.88 (0.51)	3.87 (0.50)	3.95 (0.53)	3.78 (0.58)	3.86 (0.53)
Calcium (mmol/L)	Male	1.34 (0.03)	1.34 (0.03)	1.34 (0.03)	1.32 (0.03)	1.28 (0.02)	1.28 (0.02)
	Female	1.41 (0.03)	1.41 (0.03)	1.39 (0.04)	1.37 (0.04)	1.35 (0.02)	1.34 (0.02)

G1(0): Group 1 (Test Dose: 0 mg/kg), G2(500): Group 2 (Test Dose: 500 mg/kg), G3(1000): Group 3 (Test Dose: 1000 mg/kg), G4(2000): Group 4 (Test Dose: 2000 mg/kg), G1R(0): Control Recovery Group (Test Dose: 0 mg/kg), G4R(2000): High Dose Recovery Group (Test Dose: 2000 mg/kg). SD: Standard Deviation.

The hormone analysis conducted in treated groups and Control, Recovery Control and High dose recovery groups did not exhibit any changes due to test item related effects in T3, T4, TSH respectively (Table 5).

**Table 5:** Hormone analysis.

Group (Dose)	Sex	T3 (pg/ml)	T4 (ng/ml)	TSH (µg/ml)
		*Mean (SD)*	*Mean (SD)*	*Mean (SD)*
G1(0)	Male	1097.54 (110.32)	58.01 (6.19)	1.05 (0.16)
	Female	992.9 (111.25)	57.09 (4.43)	1 (0.12)
G2(500)	Male	1134.48 (146.76)	58.17 (5.75)	1.05 (0.22)
	Female	1039.24 (61.92)	59.38 (5.44)	0.98 (0.12)
G3(1000)	Male	1185.43 (135.12)	59.03 (5.71)	1.01 (0.12)
	Female	1045.28 (79.1)	59.21 (5.09)	1 (0.16)
G4(2000)	Male	1226.85 (95.42)	59.65 (4.58)	1.08 (0.19)
	Female	1094.15* (66.57)	60.23 (4.92)	1 (0.14)
G1R(0)	Male	1089.98 (128.17)	57.15 (5.06)	1.14 (0.15)
	Female	1094.23 (93.48)	53.68 (4.25)	1.06 (0.09)
G4R(2000)	Male	1138.6 (128.09)	60.61 (9.1)	1.16 (0.08)
	Female	1204.33 (131.4)	58.45 (4.71)	1.1 (0.11)

G1(0): Group 1 (Test Dose: 0 mg/kg), G2(500): Group 2 (Test Dose: 500 mg/kg), G3(1000): Group 3 (Test Dose: 1000 mg/kg), G4(2000): Group 4 (Test Dose: 2000 mg/kg), G1R(0): Control Recovery Group (Test Dose: 0 mg/kg), G4R(2000): High Dose Recovery Group (Test Dose: 2000 mg/kg). SD: Standard Deviation.

* Statistically significant increase than G1 (p value ≤ 0.05).

### 3.8 Pathology, organ weight and histopathology

#### 3.8.1 External

No external gross pathological findings were observed in any of the animals in treated groups and control group animals. Table 6.

**Table 6:** Summary of gross pathology.

Group (Dose) (mg/kg b.wt.)	G1 (0)		G2 (500)		G3 (1000)		G4 (2000)		G1R (0)		G2R (2000)	
Sex	M	F	M	F	M	F	M	F	M	F	M	F
No. of animals per group	10	10	10	10	10	10	10	10	5	5	5	5
No. of animals found dead	0	0	0	0	0	0	0	0	0	0	0	0
**Gross Pathology Observations**												
**External Lesions**												
NAD	10	10	10	10	10	10	10	10	5	5	5	5
**Internal Lesions**												
Spleen Enlarged	2	2	0	0	0	0	0	0	0	2	0	0
Uterus distended with clear fluid	NA	5	NA	2	NA	2	NA	3	NA	0	NA	2

G1(0): Group 1 (Test Dose: 0 mg/kg), G2(500): Group 2 (Test Dose: 500 mg/kg), G3(1000): Group 3 (Test Dose: 1000 mg/kg), G4(2000): Group 4 (Test Dose: 2000 mg/kg), G1R(0): Control Recovery Group (Test Dose: 0 mg/kg), G4R(2000): High Dose Recovery Group (Test Dose: 2000 mg/kg). NAD: No abnormality detected; NA: Not applicable.

#### 3.8.2 Internal

No test item related internal gross pathological findings were observed in any of the treated groups when compared with control. All other gross pathological findings observed were either related to physiological, to spontaneous or incidental changes.

No test item related gross pathological findings were observed in any organs or tissues with the control, control recovery, treated and high dose recovery groups animals.

#### 3.8.3 Absolute organ weight

No test item related statistically significant changes were observed in the absolute organ weights of both male and female animals of test item exposed groups of main and recovery group when compared with concurrent control. Whereas significant changes were observed in mean absolute uterus with cervix weight in females of the G4R group and was considered physiological as the changes were not observed in G4 (test item high dose) group animals when compared to control group G1R also, the changes were not considered toxicologically significant. Hence organ weight changes observed were not considered as test item related effect.

#### 3.8.4 Relative organ weight

No test item related statistically significant changes were observed in the relative organ weights of both male and female animals of test item exposed groups of main and recovery group when compared with concurrent control. Whereas significant changes were observed in mean relative spleen and thyroids with parathyroid weight in females of the G3 group, was considered incidental as the changes were unisex, and were not observed in G4 (test item high dose) group animals, the changes were not considered toxicologically significant. Hence organ weight changes observed were not considered as test item related effect.

### 3.9 Histopathology

**Fig. 3:**
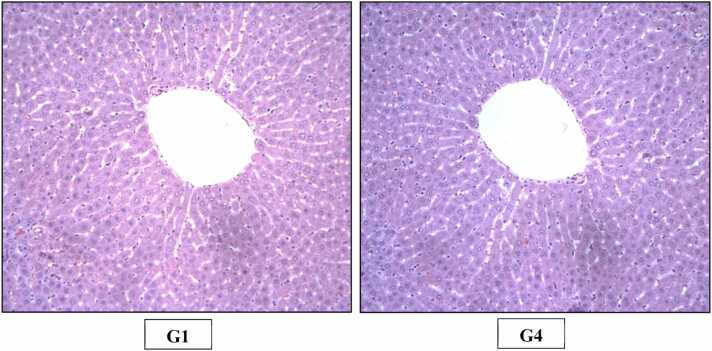
H&E staining of rats’ Liver (X200). Photomicrograph of Liver of Control (G1) and High dose (G4) group showing normal histological appearance of hepatocytes, central vein and sinusoid.

**Fig. 4:**
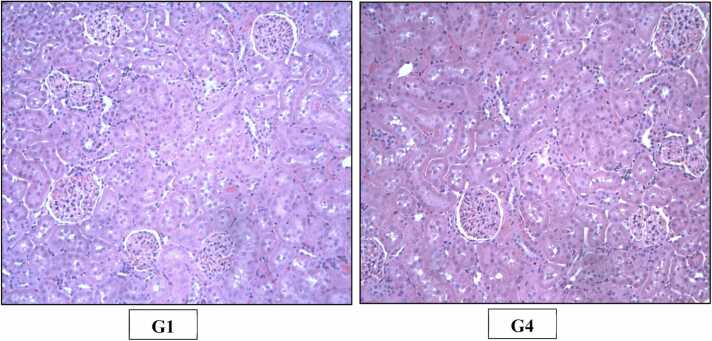
H&E staining of rats’ Kidney (X200). Photomicrograph of Kidney of Control (G1) and High dose (G4) group showing normal histological appearance of renal tubules and glomeruli.

**Fig. 5:**
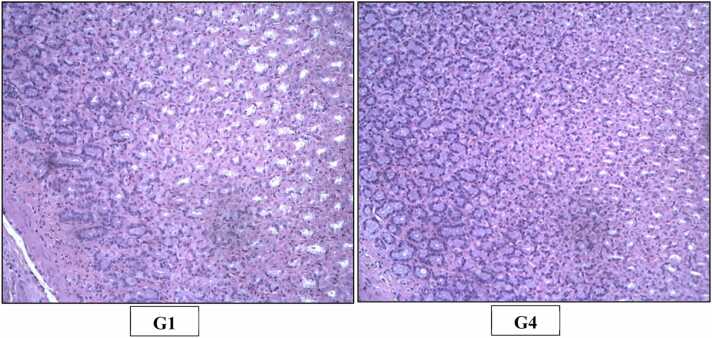
H&E staining of rats’ Stomach (X200). Photomicrograph of Stomach of Control (G1) and High dose (G4) group showing normal histological appearance of glandular stomach.

**Fig. 6:**
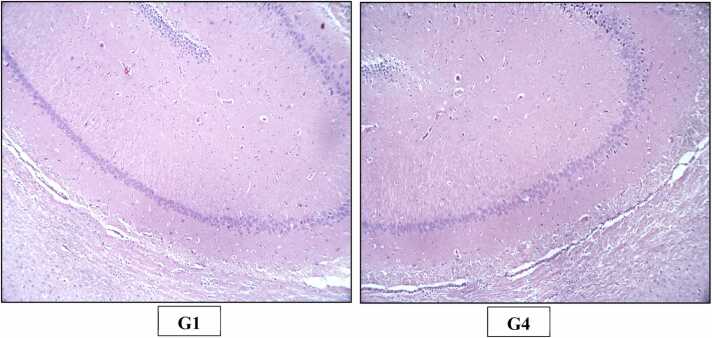
H&E staining of rats’ Brain (X100). Photomicrograph of Brain of Control (G1) and High dose (G4) group showing normal histological appearance of hippocampus.

**Fig. 7:**
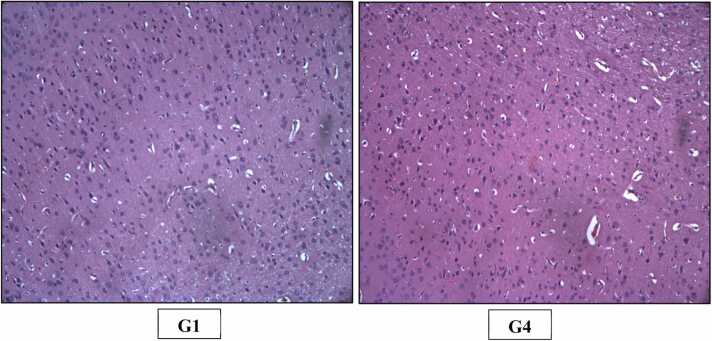
H&E staining of rats’ Brain (X200). Photomicrograph of Brain of Control (G1) and High dose (G4) group showing normal histological appearance of cerebral cortex.

**Fig. 8:**
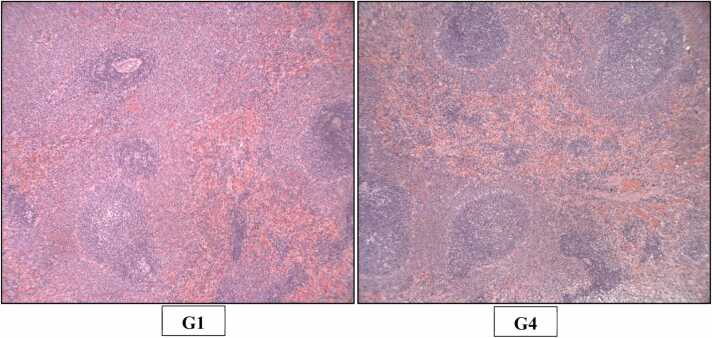
H&E staining of rats’ Spleen (X100). Photomicrograph of Spleen of Control (G1) and High dose (G4) group showing normal histological appearance of red pulp, white pulp and lymphoid nodules.

**Fig. 9:**
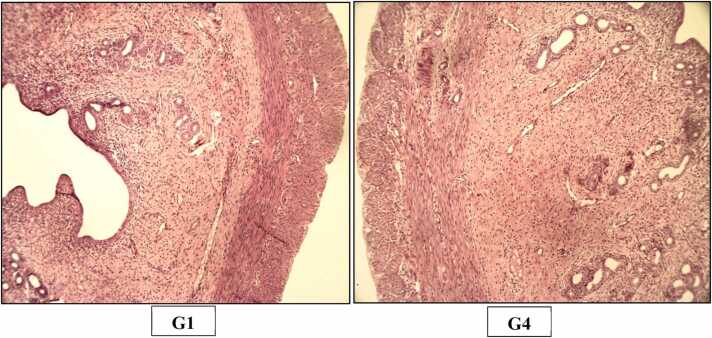
H&E staining of rats’ Uterus (X100). Photomicrograph of uterus of Control (G1) and high dose (G4) group showing normal histological appearance of Endometrium. Endometrium consisted of columnar epithelium and a lamina propria. The lamina propria was intact with a rich glandular component and highly cellular stroma.

The histopathological examination in the study was done as per goRENI (International Harmonization of Nomenclature and Diagnostic criteria - INHAND) using grading system comprised of within normal limits, minimal, mild, moderate, marked and severe [15]. Microscopic examination did not reveal any test item related histopathological finding in any of animals of high dose group when compared with animals of control group (Fig. 3, Fig. 4, Fig. 5, Fig. 6, Fig. 7, Fig. 8, Fig. 9).

Findings recorded were neither dose dependent nor any increase in incidence / severity was observed, when compared to concurrent control. All other histopathological findings observed were either related to physiological, to congenital, and spontaneous or were incidental and routinely observed in rat of this age. No signs of delayed toxicity were also observed after treatment period for 14 days.

• In histopathology, no test item related lesions or findings were recorded in high dose main group animals when compared to control animals.
• There were no significant changes in the satellite group during and post treatment observation. No signs of delayed toxicity were also observed after treatment period for 14 days.

In an acute toxicity study, oral LD50 of withania somnifea extract in Wistar rats was found to be greater than 2000 mg/kg body weight. Compared to the control group in sub-acute toxicity study, administration of extract did not show any toxicologically significant treatment related changes in clinical observations, ophthalmic examination, body weight gain, feed consumption, clinical pathology evaluation, and organ weight. Hematological and serum chemistry parameters were found to be within the normal limits. Terminal necropsy did not reveal any treatment related gross or histopathological findings [14]. Our repeated dose toxicity study findings were similar to this acute and sub-acute toxicity findings and adds crucial exploratory evidence to toxicity profile of Withania somnifera - ashwagandha.

## 4 Conclusions

The test item KSM-66 Ashwagandha Root Extract treated dose levels of 500, 1000 and 2000 mg/kg body weight did not exhibit any morbidity/mortality or untoward clinical signs of toxicity or produce any test item related effect in weekly body weight, body weight changes, feed consumption, haematology, and biochemical parameters. There were no test item related effects in thyroid hormone levels and histopathological examination of liver did not reveal any lesions or findings in high dose main group animals when compared to control animals. Under the tested conditions of the study and based on the toxicological endpoints evaluated, during the experimental and recovery period, the test item KSM-66 Ashwagandha root extract was found to be well tolerated up to 2000 mg/kg/body weight, when administered orally for a period of 90 days. Hence the No Observed Adverse Effect Level (NOAEL) of KSM-66 Ashwagandha Root Extract was found to be 2000 mg/kg/body weight by oral route in rats.

## Authors contribution

Authors PK, RS and VG contributed towards the conduct of the study, other study activities, data collection and analysis. DL contributed towards the study design, data review and manuscript preparation.

## Funding

This research was supported by Shri Kartikeya Pharma, Telangana, India.

## CRediT authorship contribution statement

**Dr. P. Kalaivani:** Investigation, Resources, Data collection and Formal Analysis, Writing – review & editing. **Dr. R. Siva:** Investigation, Resources, Data collection and Formal analysis, Writing – review & editing. **Dr. V. Gayathri:** Investigation, Resources, Data collection and Formal analysis, Writing – review & editing. **Dr. Deepak Langade:** Conceptualization, Methodology, Data curation, Writing – original draft, Writing – review & editing.

## Declaration of Competing Interest

The authors declare the following financial interests/personal relationships which may be considered as potential competing interests: Dr. P. Kalaivani, Dr. R.Siva, Dr. V. Gayathri reports financial support and equipment, drugs, or supplies were provided by Shri Kartikeya Pharma, Telangana, India. Dr. P. Kalaivani, Dr. R.Siva, Dr. V. Gayathri reports a relationship with Centre For Toxicology and Developmental Research (CEFTE), Sri Ramachandra Institute of Higher Education and Research, Chennai, Tamil Nadu, India that includes: employment.

## Data Availability

The data that support the findings of this study is available from the corresponding author on reasonable request. The data is not publicly available due to privacy or ethical restrictions.
